# News and Events of the Week

**Published:** 1911-06-17

**Authors:** 


### NEWS AND EVENTS OF THE WEEK.

The China Emergency Appeal Committee asks for the sum of £100,000 to assist Christian Missions in China to take advantage of the present unparalleled openings by establishing Union Medical Training Colleges for Chinese students.

The Union Medical College at Pekin, which is the outcome of medical missionary work, and has as its teaching staff the medical missionaries of five different missionary societies, is the most complete attempt yet made in China to give a full education in Western medicine. At a recent graduation ceremony of the sixteen students who were the first to receive a diploma granted by the Imperial Board of Education, Sir John Jordan, the British Minister who presided, spoke of the valuable work done in fighting the plague, and exhorted the students to use to the full their professional skill as a valuable asset in the advancement of the great empire.

The King has been graciously pleased to sanction the following promotions in and appointments to the Order of the Hospital of St. John of Jerusalem in England. As Knights of Justice (from Knights of Grace), H.E. the Lord Islington of Islington, K.C.M.G., D.S.O.; Colonel Sir James Richardson Andrew Clark, Bart., C.B. As Knight of Grace, the Right Hon. the Earl of Arran, K.P. As Ladies of Grace, the Right Hon. the Lady Llangattock and her Grace the Duchess of Hamilton. As Esquire, Lieut. Wm. R. Gatt, R.M.A.

We have received a copy of the fourth edition of Merck's "Manual of the Materia Medica," published by the well-known American firm, Merck and Co., of New York. It consists of a small volume of 493 pages divided into four sections. The first is devoted to materia medica, the second to indications, the third to a classification of medicaments according to their physiological action, and the fourth to a miscellany including the treatment of poisoning, a posological table, the analysis of urine and tables of obstetric dates and of the eruptive fevers. While intended mainly for distribution in the United States, the publishers have set aside a limited number of copies to supply requests from members of the medical and pharmaceutical professions in other English-speaking countries. Those wishing to procure a copy of the work should forward a postal money order for 1s. 6d. or 35 cents to Merck and Co., Manufacturing Chemists, New York.

